# Lung Ultrasound Monitoring of Legionella Ventilator-Associated Pneumonia in an Extremely Low-Birth-Weight Infant

**DOI:** 10.3390/diagnostics12092253

**Published:** 2022-09-18

**Authors:** Jing Liu, Ru-Xin Qiu

**Affiliations:** 1 Department of Neonatology and NICU, Beijing Chao-Yang Hospital, Capital Medical University, Beijing 100043, China; 2 Department of Neonatology and NICU, Beijing Chao-Yang District Maternal and Child Healthcare Hospital, Beijing 100021, China

**Keywords:** lung ultrasound, ventilator-associated pneumonia, Legionella pneumophila, pneumonia, newborn infant, extremely low birth weight

## Abstract

Ventilator-associated pneumonia (VAP) is a common complication of different severe lung diseases that need to be treated with mechanical ventilation in newborn infants. However, VAP due to Legionella pneumophila infection is rarely reported in the literature, especially in extremely low-birth-weight (ELBW) infants. Lung ultrasound (LUS) has been used in the diagnosis of neonatal pneumonia, but there is no literature on the ultrasound characteristics of Legionella-VAP in ELBW infants. This paper introduced the typical LUS findings of Legionella-VAP in ELBW infants, which mainly includes severe and large-area lung consolidation and atelectasis in the bilateral lungs; whether there is blood supply in the consolidated area has an important reference value for predicting the prognosis. In addition, the treatment and management experience were also introduced together, thereby helping us to deepen the understanding of the disease and avoid missed diagnoses.

**Figure 1:**
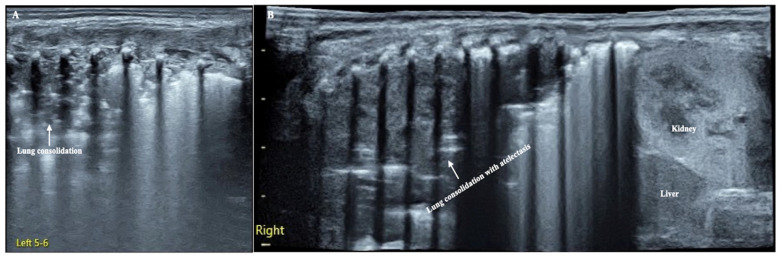
LUS manifestation of the patient. (**A**): Left lung. Large-area lung consolidation with jagged boundaries (mainly involved the upper lung field), while in the lower lung field, lung edema and subpleural consolidation were predominant, which indicate the presence of pneumonia [1]. (**B**): Right lung (extended view). Lung consolidation was almost involved in the whole lung field with regular edges, and there was almost no bronchial aeration in the consolidation area, which means that the entire right lung was atelectatic [2].

The patient was a male premature infant delivered by cesarean section because of placental abruption at gestational age 28 weeks with a birth weight of 900 g. The infant was hospitalized at the NICU due to severe respiratory distress and subsequently complicated with diffuse intravascular coagulation (DIC) at the late stage of hyperfibrinolysis. A blood examination showed a white blood cell count (WBC) of 29.3 × 10^9^/L and a c-reactive protein (CRP) of >150 mg/L (normally, <2.0 mg/L) at 4 h after birth. The patient’s condition stabilized after 10 days of invasive mechanical ventilation, anti-DIC and broad-spectrum antibiotic treatment. However, from the 14th day after birth, the infant had persistent fever (a temperature higher than 38 °C), and dyspnea reoccurred. The fever lasted for more than two weeks with a temperature >38 °C (highest 38.8 °C); the WBC count was elevated between a total of (21~29) × 10^9^/L with a significantly elevated neutrophil ratio (>80%); the CRP increased (>150 mg/L for more than 2 weeks) for more than three weeks; the platelet count continued to decrease for more than 2 weeks (minimum <10 × 10^9^/L); and three blood cultures and three deep sputum cultures and peripherally inserted central catheter (PICC) tip cultures showed no pathogenic bacterial growth. An LUS examination showed a large area of lung consolidation forming significant atelectasis in both lungs. The left lung mainly involved the upper lung field, while the right lung was almost consolidated in all lung fields (Figure 1). There was almost no bronchial inflation in the consolidation area, but a Doppler ultrasound showed that blood supply was still present well in the consolidated lung fields (Figure 2). This is very different from the normal lung images, which present as a bamboo sign on a B-mode ultrasound (Figure 3). During this period, the infant successively received meropenem, imipenem, linezolid, the fourth generation of cephalosporin, metronidazole and other antibiotics, and his condition still did not improve. Although both mycoplasma and chlamydia antibody/antigen tests were negative, when the antibiotics were adjusted to macrolide antibiotics (azithromycin) on day 28 postnatally, the temperature of the infant fell below 38 °C that day, and then, the temperature remained normal. Then, with the informed consent of the parents, blood samples were collected for metagenomic next-generation sequencing (mNGS) testing for pathogens, and the detection result confirmed legionella pneumophila infection. Therefore, the patient continued to receive azithromycin. The temperature remained normal, and the WBC, platelet and CRP levels gradually returned to the normal range within a week. The LUS showed that the scope of the lung consolidation gradually narrowed until it disappeared completely after 20 days (Figure 4). The total treatment period lasted for nearly 4 weeks, and the patient was discharged on the 70th day after birth, with a weight of 2620 g at that time. The infant was followed up for nearly 12 months and experienced normal growth. Over the past decade, LUS has been widely used in the diagnosis and differential diagnosis of neonatal lung diseases [3,4], including neonatal pneumonia [5,6,7]. LUS has a number of advantages over CXR; it should serve as a complementary diagnostic method in providing accurate, timely and reliable information [8,9]. The major finding of the LUS examination in this patient was the large-area consolidation in the bilateral lungs. There were a few air bronchograms in the consolidation area of the left lung but no air in the consolidated area of his whole right lung, which meant serious atelectasis (Figure 1). However, Doppler ultrasonography showed that there was still good blood supply in the lung tissues with consolidation and atelectasis (Figure 2). The presence of blood supply is a prerequisite for the consolidation and atelectasis of lung tissue to recover to normal [2]. 

Legionella exists in soil and sewage and is an aerobic Gram-negative bacterium. In soil and aquatic systems, L. pneumophila can invade and survive intracellularly in various protozoans [10]. L. pneumophila infection is acquired in the community, but cases and outbreaks of hospital acquisition from hot water systems have become increasingly common [11,12,13]. Legionella pneumonia is most common in middle-aged men and rarely occurs in newborn infants; thus far, only a few dozen neonatal cases have been reported, and most of them are late preterm infants and term infants, while extremely premature infants and ELBW infants are rarely included [14,15]. Water births or drinking contaminated water may be the main cause of neonatal LP infection [13,16,17,18,19]. We identified L. pneumophila in the infant’s blood sample and deep sputum sample by mNGS; therefore, we believe that this infant may have had VAP caused by L. pneumophila infection. The mortality rate of neonatal LP is approximately 50%; although the baby was of very small gestational age and very low birth weight as well as complicated with systemic multiple organ failure, he was eventually cured with effective antibiotics and ventilator treatment, assisted by bronchoalveolar lavage to improve his lung re-expansion [20,21]. In general, the diagnosis of legionella pneumonia in newborns is very difficult. According to the clinical history of this patient and the literature reports, the possibility of Legionella infection should be considered when the infant experiences the following situations during hospitalization. (1) After the original disease is cured or improved, the infant’s condition repeats again with fever, severe dyspnea and other clinical manifestations. (2) Conventional broad-spectrum strong antibiotics, such as meropenem, imipenem and other treatments, are ineffective, and the patient’s condition deteriorates rapidly. (3) Macrolide antibiotics are effective in treatment. Under such conditions, timely etiological monitoring and even mNGS tests are needed to confirm the diagnosis. This case also confirmed that LUS was helpful in finding the lung lesions. However, because this kind of pneumonia is so rare, especially in very premature infants, it is difficult to systematically study its ultrasound findings. In addition, the progressive exacerbation of lung disease due to a lack of timely and effective treatment may be one of the reasons for the extensive consolidation and severe atelectasis. Meanwhile, other types of bacterial pneumonitis are sensitive to commonly used antibiotics, respond well to treatment and rarely accumulate atelectasis throughout the lungs, although they also show consolidation on ultrasound [1,5,6,7]. The problems may be as following: (1) LUS may well detect atelectasis, infiltrations, pneumothorax, etc. However, different bacterial pathogens may cause very similar sonographic pictures; therefore, LUS is not usually able to specify the causative agent of an infiltration or atelectasis. (2) To fully master LUS technology is not an easy thing and for which one needs to go through long-term research and become fully experienced. Several guidelines have been published to facilitate the study of LUS [22,23,24].

## Figures and Tables

**Figure 2:**
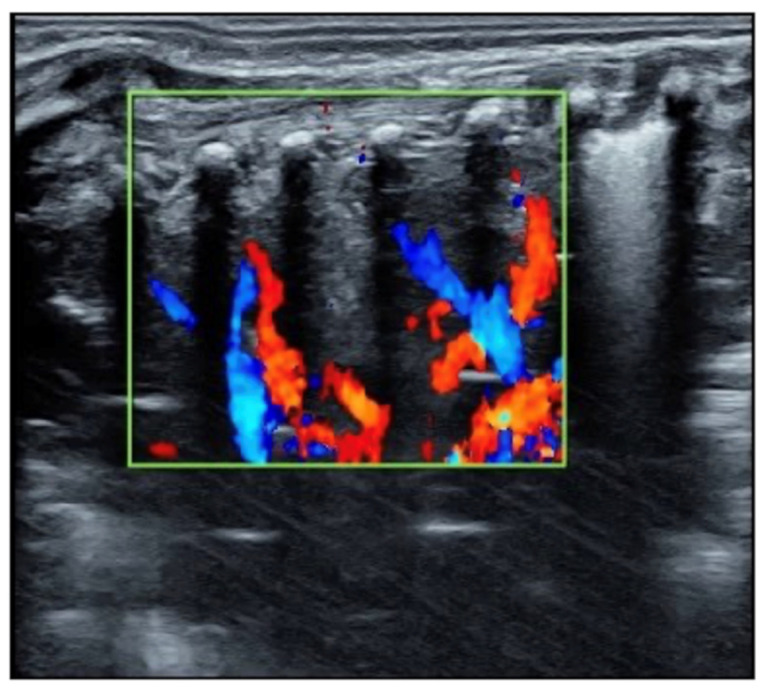
Blood supply in the consolidated lung area; Doppler ultrasound showed that the blood supply existed within the consolidated area. The presence of blood supply in the lung tissue of consolidation is a prerequisite for the recovery of severe atelectasis.

**Figure 3:**
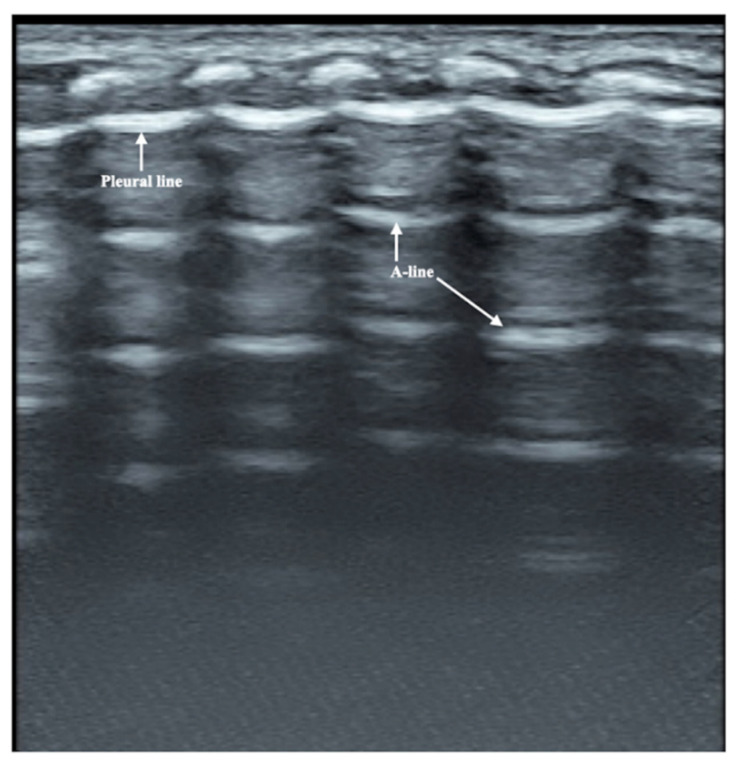
Normal lung ultrasound manifestations. On B-mode ultrasound, the pleural line and A-line show smooth, regular and hyperechoic lines arranged in parallel and equidistant from each other. Together, they form a kind of bamboo-like performance, which is known as the bamboo sign.

**Figure 4:**
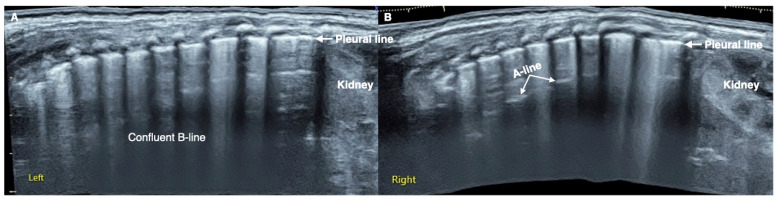
The convalescent ultrasound findings of this L. pneumoniae. LUS (extended view) showed significant confluent B-lines in both lung fields ((**A**): left lung, (**B**): right lung), the pleural line is rough and fuzzy, A-line is present only in a few intercostal spaces of the right lung (**B**). Lung consolidation and atelectasis disappeared in the whole lung fields. It is suggested that there is obvious pulmonary edema in the infant’s lungs.

## Data Availability

Not applicable.

## Abbreviations

VAP	ventilator-associated pneumonia.
EPI	extremely premature infant.
ELBW	extremely low birth weight.
LUS	lung ultrasound.
LP	legionella pneumonia.
DIC	diffuse intravascular coagulation.
WBC	white blood cell count.
CRP	c-reactive protein.
PICC	peripherally inserted central catheter.
mNGS	metagenomic next-generation sequencing.
L. pneumoniae	legionella pneumophila.
CXR	X-ray.
